# Supplementary material for: Grading by size to reduce the opportunity for domestication selection in hatchery-reared steelhead (Oncorhynchus mykiss)
Source: PLoS One. 2025 Dec 16;20(12):e0338623. doi: 10.1371/journal.pone.0338623 (PMC12707659; doi:10.1371/journal.pone.0338623)
Supplement: Attachment — Submitted filename: PONE-D-25-01027.pdf [file pone.0338623.s002.pdf]

PLOS ONE

Grading by size to reduce the opportunity for domestication selection in hatchery-reared steelhead (<i>Oncorhynchus mykiss</i>)

--Manuscript Draft--

**Manuscript Number:**	PONE-D-25-01027
**Article Type:**	Research Article
**Full Title:**	Grading by size to reduce the opportunity for domestication selection in hatchery-reared steelhead (<i>Oncorhynchus mykiss</i>)
**Short Title:**	Grading and steelhead (<i>Oncorhynchus mykiss</i>) domestication
**Corresponding Author:**	Miriam Obley, MS<br>Oregon State University<br>Corvallis, Oregon UNITED STATES OF AMERICA
**Keywords:**	Hatchery; Domestication; relative reproductive success; salmon; fitness
**Abstract:**	Fish that are produced in hatcheries often produce fewer surviving adult offspring than do wild fish when both spawn in the wild. This difference in fitness is likely due to inadvertent selection for adaptation to the hatchery environment. Size at release is positively correlated with survival at sea. Therefore, selection should favor traits that promote fast growth in the hatchery even if those traits are maladaptive in the wild. In that case, changing hatchery conditions to reduce the variance in size at release would reduce the opportunity for domestication selection. Here we test whether grading by size and raising each size group separately can substantially reduce the variance in size at release. We graded a mix of 15 full-sibling families of winter run steelhead (<i>Oncorhynchus mykiss</i>) juveniles into small, medium, and large body size groups. We then promoted growth in the initially-small fish with excess food and low densities, and slowed growth in the initially-large fish with restricted feedings and high densities. We successfully held back the growth of the initially-large fish, but the initially-small fish never caught up with the controls, despite being raised under ideal conditions. This result suggests that inherent physiological or behavioral factors, rather than interactions with larger fish, restrict the growth of small fish. Both the total variance among individual fish and the variance among family means was reduced by the grading treatment, although only the reduction in total variance among individuals was statistically significant.<br>The reduced variance due to slowing the growth of the initially-large fish indicates that grading could possibly be a tool used by hatcheries to lessen selection on salmonids. However, it would first be necessary to better understand the factors limiting growth in the smaller fish.
**Order of Authors:**	Miriam Obley
	Stephanie R. Bollmann
	Ruth Milston-Clements
	Michael Blouin
**Additional Information:**	
**Question**	**Response**
**Financial Disclosure**<br><br>Enter a financial disclosure statement that describes the sources of funding for the work included in this submission. Review the submission guidelines for detailed requirements. View published research articles from *PLOS ONE* for specific examples.	Yes

Powered by Editorial Manager® and ProduXion Manager® from Aries Systems Corporation

This statement is required for submission and **will appear in the published article** if the submission is accepted. Please make sure it is accurate.<br><br>**Funded studies**<br>Enter a statement with the following details:<br>• Initials of the authors who received each award<br>• Grant numbers awarded to each author<br>• The full name of each funder<br>• URL of each funder website<br>• Did the sponsors or funders play any role in the study design, data collection and analysis, decision to publish, or preparation of the manuscript?<br><br>Did you receive funding for this work?	
Please add funding details.<br>as follow-up to "**Financial Disclosure**<br><br>Enter a financial disclosure statement that describes the sources of funding for the work included in this submission. Review the submission guidelines for detailed requirements. View published research articles from *PLOS ONE* for specific examples.<br><br>This statement is required for submission and **will appear in the published article** if the submission is accepted. Please make sure it is accurate.<br><br>**Funded studies**<br>Enter a statement with the following details:<br>• Initials of the authors who received each award<br>• Grant numbers awarded to each author<br>• The full name of each funder<br>• URL of each funder website<br>• Did the sponsors or funders play any role in the study design, data collection and analysis, decision to publish, or preparation of the manuscript?	This research was supported by the Oregon Hatchery Research Center (https://www.dfw.state.or.us/fish/ohrc/). The funders had no role in study design, data collection and analysis, decision to publish, or preparation of the manuscript.

Did you receive funding for this work?"	
Please select the country of your main research funder (please select carefully as in some cases this is used in fee calculation).<br>as follow-up to "**Financial Disclosure**<br><br>Enter a financial disclosure statement that describes the sources of funding for the work included in this submission. Review the submission guidelines for detailed requirements. View published research articles from *PLOS ONE* for specific examples.<br><br>This statement is required for submission and **will appear in the published article** if the submission is accepted. Please make sure it is accurate.<br><br>**Funded studies**<br>Enter a statement with the following details:<br>• Initials of the authors who received each award<br>• Grant numbers awarded to each author<br>• The full name of each funder<br>• URL of each funder website<br>• Did the sponsors or funders play any role in the study design, data collection and analysis, decision to publish, or preparation of the manuscript?<br><br>Did you receive funding for this work?"	UNITED STATES - US
**Competing Interests**<br><br>Use the instructions below to enter a competing interest statement for this submission. On behalf of all authors, disclose any competing interests that could be perceived to bias this work—acknowledging all financial support and any other relevant financial or non-financial competing interests.<br><br>This statement is required for submission	The authors have declared that no competing interests exist.

Powered by Editorial Manager® and ProduXion Manager® from Aries Systems Corporation

and **will appear in the published article** if the submission is accepted. Please make sure it is accurate and that any funding sources listed in your Funding Information later in the submission form are also declared in your Financial Disclosure statement.<br><br>View published research articles from *PLOS ONE* for specific examples.<br><br>**NO authors have competing interests**<br><br>Enter: *The authors have declared that no competing interests exist.*<br><br>**Authors with competing interests**<br><br>Enter competing interest details beginning with this statement:<br><br>*I have read the journal's policy and the authors of this manuscript have the following competing interests: [insert competing interests here]*<br><br>* typeset	
**Ethics Statement**<br><br>Enter an ethics statement for this submission. This statement is required if the study involved:<br><br>• Human participants<br>• Human specimens or tissue<br>• Vertebrate animals or cephalopods<br>• Vertebrate embryos or tissues<br>• Field research<br><br>Write "N/A" if the submission does not require an ethics statement.<br><br>General guidance is provided below. Consult the submission guidelines for detailed instructions. **Make sure that all information entered here is included in the Methods section of the manuscript.**	Animal Care and Use Protocol IACUC-2021-0236<br>MS222 was used for anesthesia and euthanasia

**Format for specific study types**<br><br>**Human Subject Research (involving human participants and/or tissue)**<br>• Give the name of the institutional review board or ethics committee that approved the study<br>• Include the approval number and/or a statement indicating approval of this research<br>• Indicate the form of consent obtained (written/oral) or the reason that consent was not obtained (e.g. the data were analyzed anonymously)<br><br>**Animal Research (involving vertebrate animals, embryos or tissues)**<br>• Provide the name of the Institutional Animal Care and Use Committee (IACUC) or other relevant ethics board that reviewed the study protocol, and indicate whether they approved this research or granted a formal waiver of ethical approval<br>• Include an approval number if one was obtained<br>• If the study involved *non-human primates*, add *additional details* about animal welfare and steps taken to ameliorate suffering<br>• If anesthesia, euthanasia, or any kind of animal sacrifice is part of the study, include briefly which substances and/or methods were applied<br><br>**Field Research**<br><br>Include the following details if this study involves the collection of plant, animal, or other materials from a natural setting:<br>• Field permit number<br>• Name of the institution or relevant body that granted permission	
**Data Availability**<br><br>Authors are required to make all data underlying the findings described fully available, without restriction, and from the time of publication. PLOS allows rare exceptions to address legal and ethical concerns. See the PLOS Data Policy and FAQ for detailed information.	Yes - all data are fully available without restriction

A Data Availability Statement describing where the data can be found is required at submission. Your answers to this question constitute the Data Availability Statement and **will be published in the article**, if accepted.<br><br>**Important:** Stating 'data available on request from the author' is not sufficient. If your data are only available upon request, select 'No' for the first question and explain your exceptional situation in the text box.<br><br>Do the authors confirm that all data underlying the findings described in their manuscript are fully available without restriction?	
**Describe where the data may be found in full sentences. If you are copying our sample text, replace any instances of XXX with the appropriate details.**<br><br>• If the data are **held or will be held in a public repository**, include URLs, accession numbers or DOIs. If this information will only be available after acceptance, indicate this by ticking the box below. For example: *All XXX files are available from the XXX database (accession number(s) XXX, XXX.).*<br>• If the data are all contained **within the manuscript and/or Supporting Information files**, enter the following: *All relevant data are within the manuscript and its Supporting Information files.*<br>• If neither of these applies but you are able to provide **details of access elsewhere**, with or without limitations, please do so. For example:<br><br>*Data cannot be shared publicly because of [XXX]. Data are available from the XXX Institutional Data Access / Ethics Committee (contact via XXX) for researchers who meet the criteria for access to confidential data.*<br><br>*The data underlying the results presented in the study are available from (include the name of the third party*	All data generated or analyzed during this study are available in the following public repository: https://github.com/obleym/Grading_Data_Obley.

and contact information or URL).<br>• This text is appropriate if the data are owned by a third party and authors do not have permission to share the data.<br><br>* typeset	
Additional data availability information:	

Manuscript
Click here to access/download;Manuscript;Grading_Obley_Submission_Figur

Grading by size to reduce the opportunity for domestication selection in hatchery-reared steelhead (*Oncorhynchus mykiss*)

Short title: Grading and steelhead (*Oncorhynchus mykiss*) domestication

Miriam B. Obley[1,#a*], Stephanie R. Bollmann[1], Ruth H. Milston-Clements[2,#b], and Michael S. Blouin[1]

[1] Department of Integrative Biology, Oregon State University, Corvallis, Oregon, USA
[2] Department of Microbiology, Oregon State University, Corvallis, Oregon, USA

[*]Corresponding Author e-mail: obleym@oregonstate.edu

[#a]Current address: Department of Fisheries, Wildlife, and Conservation Sciences, Oregon State University, USA
[#b]Current address: Division of Research and Innovation, Oregon State University

Abstract

Fish that are produced in hatcheries often produce fewer surviving adult offspring than do wild fish when both spawn in the wild. This difference in fitness is likely due to inadvertent selection for adaptation to the hatchery environment. Size at release is positively correlated with survival at sea. Therefore, selection should favor traits that promote fast growth in the hatchery even if those traits are maladaptive in the wild. In that case, changing hatchery conditions to reduce the variance in size at release would reduce the opportunity for domestication selection. Here we test whether grading by size and raising each size group separately can substantially reduce the variance in size at release. We graded a mix of 15 full-sibling families of winter run steelhead (*Oncorhynchus mykiss*) juveniles into small, medium, and large body size groups. We then promoted growth in the initially-small fish with excess food and low densities, and slowed growth in the initially-large fish with restricted feedings and high densities. We successfully held back the growth of the initially-large fish, but the initially-small fish never caught up with the controls, despite being raised under ideal conditions. This result suggests that inherent physiological or behavioral factors, rather than interactions with larger fish, restrict the growth of small fish. Both the total variance among individual fish and the variance among family means was reduced by the grading treatment, although only the reduction in total variance among individuals was statistically significant. The reduced variance due to slowing the growth of the initially-large fish indicates that grading could possibly be a tool used by hatcheries to lessen selection on salmonids. However, it would first be necessary to better understand the factors limiting growth in the smaller fish.

Keywords: Hatchery, domestication, relative reproductive success, salmon, fitness

Introduction

Hatchery programs have been heavily implemented in the Pacific Northwest to mitigate the decline of wild salmonid populations. While these programs are successful at producing fish for harvest, there is less evidence supporting supplementation programs on the recovery of wild populations and the success of their progeny (Waples, Ford, and Schmitt 2007; McMillan et al. 2023). In contrast, fish reared under hatchery conditions tend to have lower reproductive success when spawning in the wild than natural origin fish (Christie, Ford, and Blouin 2014; Koch and Narum 2021). This decrease in fitness is likely due to domestication (i.e. adaptation to the hatchery setting), which can lead to behavioral and behavioral and physiological differences between captive and wild populations (Howe et al. 2024; Wright et al. 2006; Pasquet 2018). However, there is a tradeoff that occurs in which the well adapted fish in the hatchery do poorly in the wild and vice versa (Christie et al. 2012). Increased growth rates in captivity have been observed in the offspring of captive-reared fish compared to the offspring of natural origin fish (Thodesen et al. 1999; Petersson and Järvi 2000; Blouin et al., 2021). Growth in the hatchery has also been linked to survival at sea because larger fish that are larger at release are more likely to return than smaller fish at release (Clarke, Flesher, and Carmichael 2014; Osterback et al. 2014; Tipping 1986).Therefore, any trait variation among fish in a hatchery that influences their growth in this novel environment may be under strong selection. Furthermore, there is substantial variation in size at release among fish reared in captivity (Feldhaus, Hoffnagle, and Carmichael 2016), family identity explains substantial variance in size among fish reared in hatcheries

(Thompson and Blouin 2015; Blouin et al. 2021), and growth in hatcheries tends to have high heritability (Carlson and Seamons 2008). Hence, there is also substantial opportunity for response to selection on whatever traits are behind variation in growth rate under hatchery conditions.

So what traits might be under selection in hatcheries? Hatchery fish tend to be more aggressive than wild fish (Weber and Fausch 2003) indicating boldness or aggression might be selected for in the hatchery environment. It is well known that individual fish vary in a correlated suite of traits that includes boldness, aggressiveness and dominance (Fu et al. 2021; Conrad et al. 2011). These “pace of life” traits tend to be consistent among individuals, highly heritable, and also correlated with physiological traits such as metabolic rate (Mazué, Dechaume-Moncharmont, and Godin 2015; Metcalfe, Van Leeuwen, and Killen 2016). Bolder, more aggressive fish gain more access to food, have a higher metabolic rate, and subsequently grow faster and more efficiently than subdominant fish reared in the same environment (Huntingford 2004; Sundström et al. 2004; Li and Brocksen, n.d.). However, excess boldness is not necessarily beneficial in the wild where other behaviors such as predator avoidance may be important to tradeoffs between growth and survival (Biro et al. 2004; Biro, Abrahams, and Post 2007).

If the wild-born offspring of hatchery fish inherit the tendency for bold/aggressive behaviors, then they might have low survival in the wild. Thus, inadvertent selection in the hatchery for heritable traits such as boldness/aggression could also explain the reduced fitness of hatchery fish when they spawn in the wild.

Aspects of fish physiology and behavior can be manipulated by changing the rearing environment (Ahlbeck Bergendahl et al. 2017). Therefore it has been hypothesized that altering environmental conditions in the hatchery could reduce the variance in size at release, thereby

reducing the opportunity for selection on whatever traits influence growth in the hatchery (Thompson and Blouin 2015). In particular, if hatchery conditions typically favor bolder/aggressive phenotypes, then introducing conditions that favor the shyer fish might reduce the variance in size at release. However, simple changes to the hatchery environment designed to favor shyer phenotypes, such as reducing rearing density, increasing water flow, or adding physical structure to tanks have achieved little reduction in the variance of fork length (Thompson and Blouin 2015; Obley et al. 2024). In general, the families that grow quickly in one environment also grow quickly in others, and vice versa for the slow-growing families. More substantial changes may need to be made in order to reduce variance in size at release.

We hypothesize that grading fish based on size (fork length), and then rearing the different size groups separately, may be one method that is more effective than small environmental changes without adding too much complexity to hatchery procedures. Grading is used in hatchery productions to create better angling opportunities, but as a result, smaller fish are often discarded (Branigan, Meyer, and Cassinelli 2021; Wallat et al. 2005). By separating large and small fish and keeping the small group, it may have the opportunity to gain access to more food, be relieved of aggressive interactions with larger (presumably bolder) fish and have the potential to grow larger and at a faster rate than they would under typical conditions (Nicieza and Metcalfe 1999; Abbott and Dill 1989). In contrast the large group's growth rate may be slowed by feed restriction so that all of the fish in the entire cohort would be more likely to reach the same target length at the time of release. Thus, grading may be an effective method to overcome inherited traits among families of hatchery-reared salmonids and therefore reduce the opportunity for inadvertent domestication selection.

Alternatively, it may be that the slower growth rate of the small fish in a hatchery setting may not result from interaction with more aggressive fish, but from inherent physiological or behavioral traits that limit their ability to take advantage of the hatchery setting. There may be some trait that the small fish have that keep them from growing rapidly in the hatchery regardless of their interaction with the larger fish. In that case, separating out the slower growing fish would have little effect on their performance.

Here we test whether grading a cohort of steelhead into fast, medium and slow growing groups, and then deliberately manipulating the target release sizes of each group via food and density, can substantially reduce the overall variance in size of the entire cohort at release.

Materials and Methods

Spawning and Incubation

All fish handling protocols were approved by the Oregon State University Institutional Animal Care and Use Committee (Animal Care and Use Protocol IACUC-2021-0236). Winter-run steelhead were collected by Oregon Department of Fish and Wildlife (ODFW) staff at the Siletz Falls Trap on the Siletz River, Oregon, and spawned by ODFW staff at the Oregon Hatchery Research Center (OHRC). Ten pairs were spawned on March 25, 2022, and six pairs on April 7, 2022 for a total of 16 individual families (A-P). Fin clips from all parents were collected and stored in 95% ethanol for future genotyping and sorting offspring into their families. All eggs were water hardened and treated in a thiamine bath for 1 hour as prophylaxis against thiamine deficiency. On both spawning dates, only one female was spawned with each male, creating 16 full sibling families that were raised in individual family trays in vertical stack incubators until they hatched and absorbed yolk sacs. The first spawn group was held on chilled water in order to sync the growth of the two groups. Fry were then transferred to separate Vexar

mesh baskets in a trough with ambient temperature water flowing through. Food was withheld for 2-3 days to avoid fungal growth. Fry were then fed and remained in the trough until they learned to feed exogenously.

Sorting into treatment tanks

On June 22, 2022, fish from each of the families were transported in coolers containing aerated river water to the John Fryer Aquatic Animal Health Lab (AAHL) at Oregon State University (approximately 1 hour drive). Upon arrival, twenty-five fish from each of the 15 families were added to each of 4 experimental tanks (n=375 fish/tank). Experimental tanks were 380 L, 3 ft diameter x 3 ft depth circular fiberglass tank with constant flow-through, UV sterilized 13 °C well water. Tanks were located outdoors and therefore received a natural photoperiod.

Project Design - Grading

Fish were fed a diet of BioOregon Bio Vita starter feed crumble and then increasing sized crumble and pellets at a frequency, pellet size and % body weight (%BW) according to ODFW feed guidelines calculated to reach target weights (supplemental material). Initially, a feed regime to reach a target release weight of 6 fish per pound (FPP) (~75g/fish) by April 14 2023 was chosen. This is in line with current hatchery practices for release size and timing of release of Winter Steelhead at the Alsea ODFW Hatchery ("ALSEA F H FALL CREEK, OREGON - Climate Summary," n.d.).

After 143 days fish had grown sufficiently such that there was a range of fork lengths (FL) within each tank. Fish from two of the four tanks were graded by measuring FL of individual fish and splitting into three groups depending on FL: large (>113 mm FL) , medium (97-113mm FL), and small (97mm FL). This resulted in 2 tanks (380 L capacity) for each FL

size bin. To create a high-density environment as an additional way to limit growth of the large fish, the fish from the two large FL tanks were then pooled into one 380 L tank (Fig 1). Two of the four original ungraded tanks remained as control tanks.

In this experimental design our goal was to compare the variance in FL of the fish that were not graded with the variance in FL of the fish that were graded, and it is fish from the same 15 families in each treatment group. In other words, at the end of the experiment there will be just two groups of fish that will be compared. One group was raised under standard (control) conditions. The other group was divided into grading size groups that were treated differently, and then combined into one group at the end of the experiment. Note that we are not using individual tanks as units of replication of a treatment, but just to increase the number of fish we could raise under each condition.

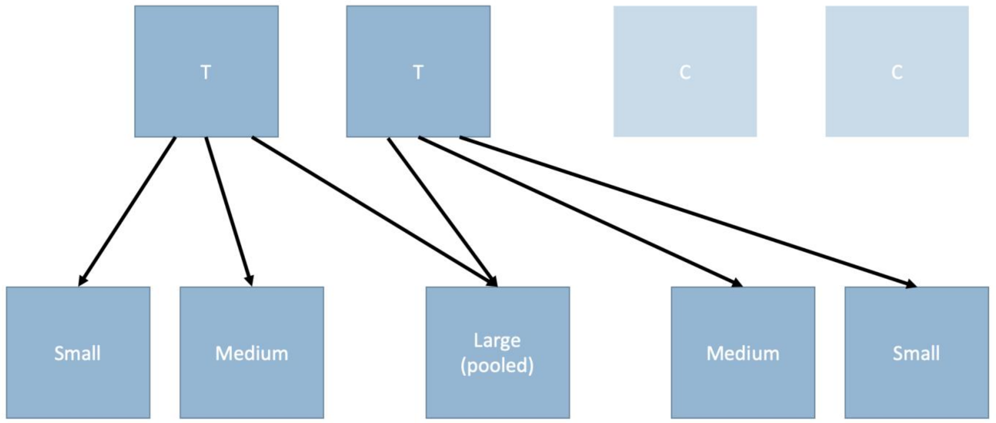

Fig 1. Two treatment tanks graded into new tanks based on fork length. T=treatment, C = control. Fish were moved into small, medium, or large tanks. The large fish were pooled into one tank to create a higher density environment. Two tanks were kept as controls. T=treatment, C = control.

At this stage, control and medium tanks continued to be fed based on % BW with the goal of reaching 6 FPP (~75g/fish) by April 14, 2023 according to ODFW feed guidelines chart.

(supplemental material) To encourage smaller fish to grow more, small tanks were fed more, with a target release weight goal of 4 FPP (~113g/fish). Conversely, the large tank was fed less, with a goal of 7 fish per lb. The large tank was also fasted two days a week to limit growth. Our goal was to have the initially-small and the initially-large groups reach the same size as the initially-medium group at the end of the experiment. Fish were sub-sampled for length and weight to adjust feed rations at least once a month during this rearing period.

Fish were euthanized on April 15, 2023 using MS222, and were measured for fork length. A fin clip from each individual fish taken and stored in 95% ethanol to preserve the DNA for genetic parentage analysis in order to be matched back to their individual families.

Health monitoring and treatments

Fish were monitored daily for health status. From July 2, 2022 to July 11, 2022, all fish were given Florfenicol medicated feed at 2% of the body weight for 10 days to treat for Bacterial Cold Water Disease (Flavobacterium psychrophilum). From August 1, 2022 – August 3, 2022 fish were given daily formalin baths to treat for the parasite Ichthyobodo necator *(Costia)*. These pathogens were present due to their initial rearing in natural river water system at the OHRC. Mortality was negligible and did not impact specific families disproportionately.

Genetic Parentage Analysis

All samples were genotyped and assigned back into their original families via genetic parentage analysis. DNA was extracted from preserved fin clips ~~w ith~~ Chelex 100 (Nelson, Beacham, and Small 1998). Six microsatellite loci (Ogo4, Omm1046, Omy7, One102, Ots4, Ssa407MP) from the SPAN B suite were multiplexed and amplified with PCR (Stephenson et al. 2009) in a single reaction. All samples were submitted to the Center for Quantitative Life Sciences Core Facilities at Oregon State University where genotype scoring was performed on

an AB 3730 capillary DNA sequencer. ABI GeneMapper® software was used to analyze the output (Applied Biosystems 3730 Genetic Analyzer, RRID:SCR_018052). Each sample was assigned back to its family using the parent genotypes and pure-exclusion parentage analysis, requiring unambiguous genotypes from each offspring for at least five loci.

Statistical Analysis: opportunity for selection

At the end of the experiment, we considered all the fish from the grading tanks to be one cohort, and all the fish from the control tanks to be another cohort. Thus, we are comparing two groups of fish, and individual fish (not tanks) are the unit of replication. We examined the effect of grading on the total variance in size (FL) among fish, and on the among-family component of variance in size. We examined the among-family component of variance because family should be a reasonable proxy for heritable variation in size (Carlson and Seamons 2008). Before examining the effect of treatment on total variance among individuals and on variance among family means, we adjusted the fork length of each individual fish to remove the random tank effects in each treatment and size group. We did this because we are interested in the total variance of fish in each group, uninflated by random tank effects. We adjusted the two control tanks to have the same mean, and each group within the grading treatment to have the same mean (i.e. the small tanks had the same mean and the medium tanks had the same mean; there was only one large tank so length was not adjusted). By adjusting length this way, the random tank effects were accounted for without removing the effect of grading. Hereafter, we use the term “treatment” to refer to the control group versus the graded group (not to the small/medium/large feeding and density regimes that were applied to the graded group).

We compared the total variance in size, and the variance among family means, between the two treatments (graded vs. control) via Fligner Killeen median tests because of the left-hand

skew in the distribution of the data (Fig. 2). To further assess the effect of treatments on the among-family variation in length, we also calculated the intraclass correlation (ICC), which is the percentage of total variation in length that is distributed among families. A higher ICC value indicates more opportunity for selection among families. So, we are testing whether the treatment significantly reduced the ICC. The ICC package "ICCest" in R was used to calculate ICC values and confidence intervals (Lessells and Boag 1987).

Results

At the end of the experiment, the total distribution of size within each treatment appeared close to normal but with a left-hand skew of very small individuals (Fig 2). Fish were 0.49 cm smaller overall in the graded cohort ($t = -5.18$, $df = 1167$, p value = 2.61e-07). From figure 3.2, it appears that this reduction in mean size results mainly from a reduction in the right-hand tail of the distribution relative to controls, with little change in the left-hand tail.

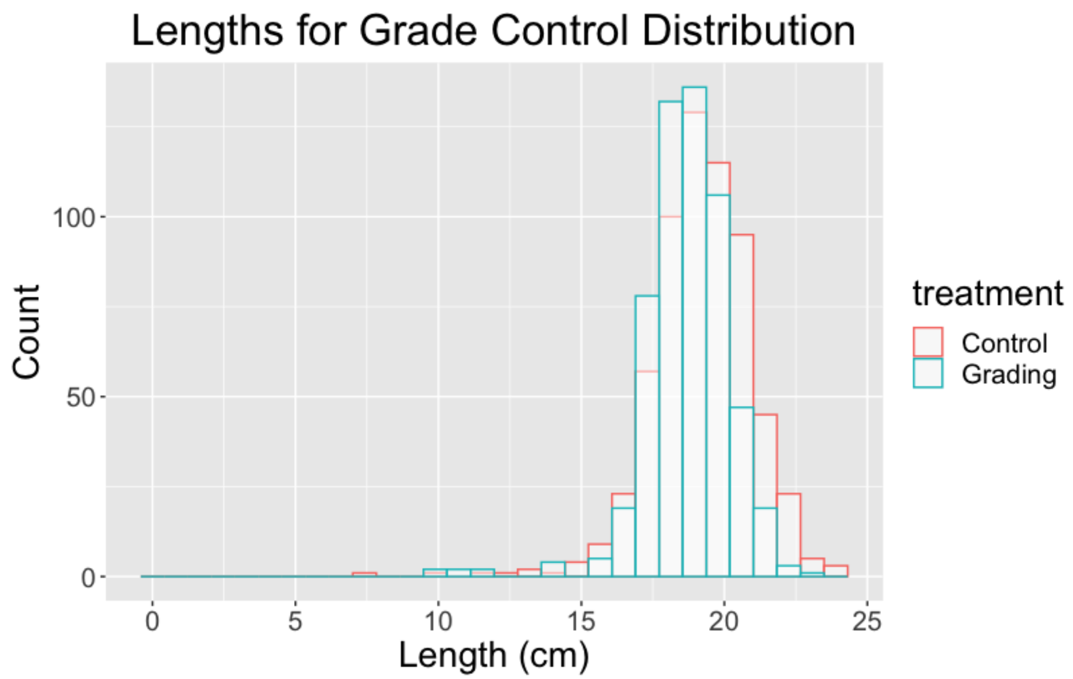

Fig 2. Histograms of body length of all the fish in each treatment. The size of each fish was adjusted to remove random tank effects within each treatment. Distributions were close to normal, but with a left-hand skew due to very small fish.

The origin of this pattern is obvious in Fig 3, which shows the average sizes of the fish in each feeding regime at the beginning and end of the experiment. The growth of the “large” grading group was successfully held back, such that at the end of the experiment they were smaller on average than the “medium” grading and control groups. However, fish in the “small” grading group never caught up, despite being raised at very low densities and being fed substantially more than the other groups (Fig 3).

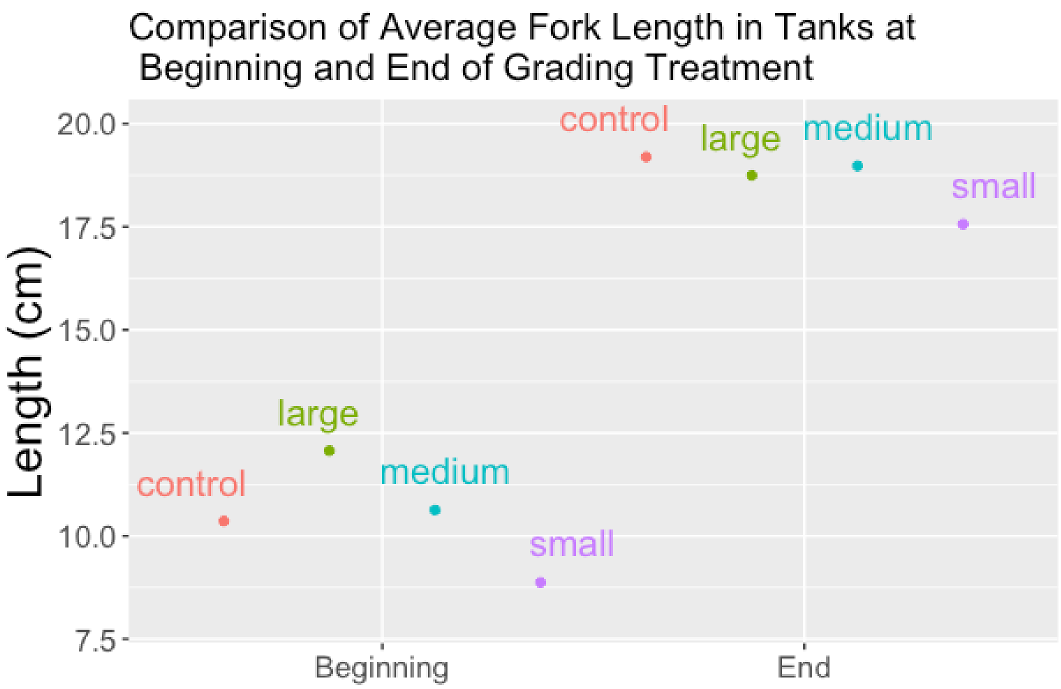

Fig 3. Comparison of mean fork length in grading and control tanks at the beginning and end of the experiment. At the end of the experiment, the initially-large grading group was smaller than the initially-medium grading and control groups. The initially-small grading group did not catch up to the other groups.

We can examine the effect of the grading treatment on the overall variance in size, on the among-family variance in size, and on the sizes of individual families. As expected, families were unequally distributed throughout the small, medium, and large grading tanks at the start of

the experiment (Fig 4). For example, there were very few members of families D, G, K, N, O, and no members of family M, represented in the initially-small grading tanks.

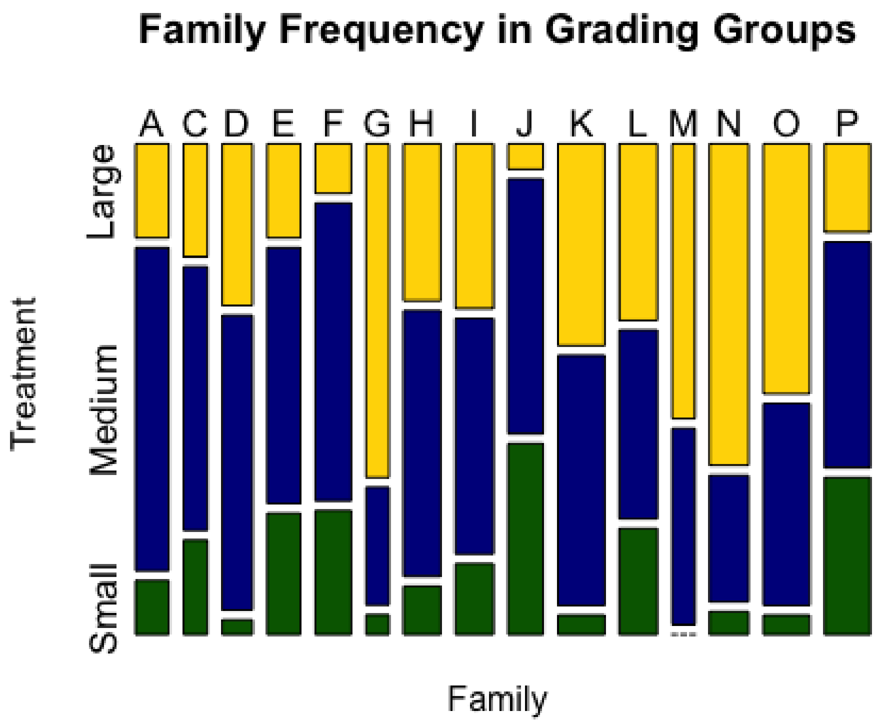

Fig 1. Distribution of families throughout the grading tanks. Families were unequally represented in each grading regime. Letters (columns) represent individual families, and the stacked bar charts show the percentage of each family that wound up in the initially-small (green), initially-medium (blue) and initially-large (yellow) groups.

At the end of the experiment, the average sizes of these initially-larger families were substantially reduced in the graded cohort relative to in the control cohort (Fig 5). While the spread of family means in the grading treatment is reduced, most of this reduction appears to result from holding back the initially fast-growing families, rather than improving the growth of the initially-slow-growing families (Fig 5).

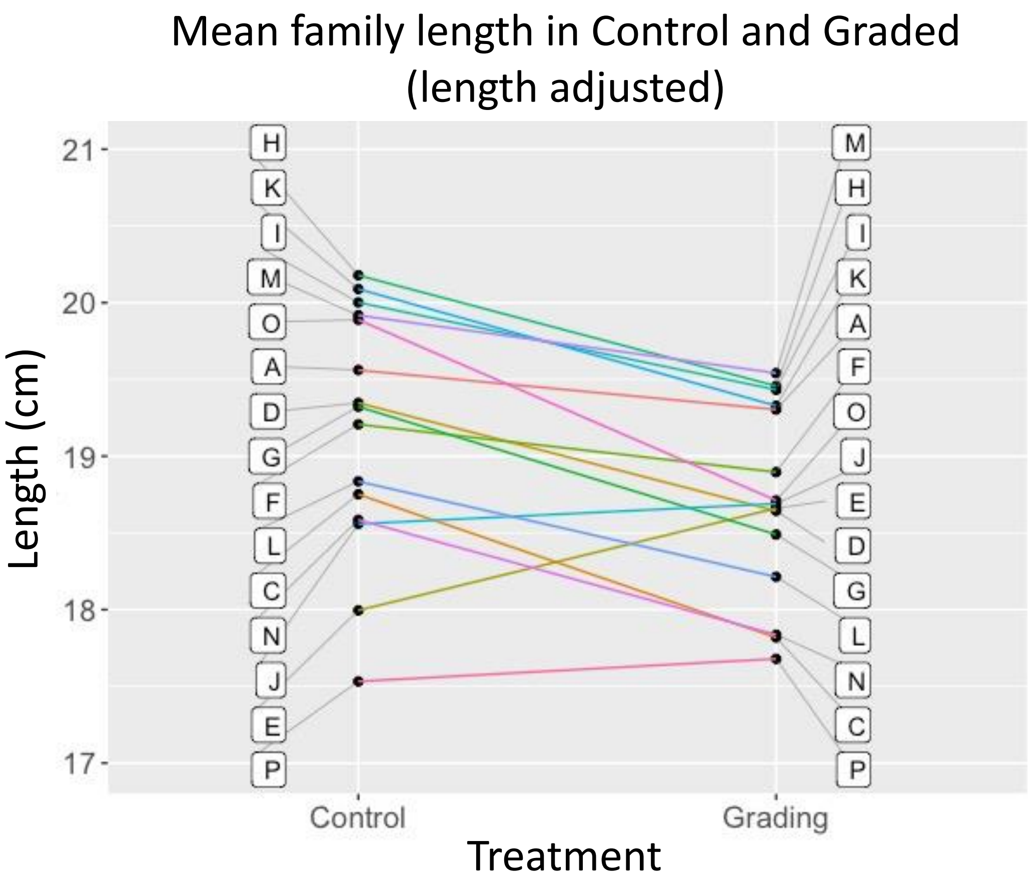

Fig 5. Comparing family mean fork lengths between control and grading treatments. The larger families had substantially reduced means in the grading treatment, which resulted from their overrepresentation in the initially-large treatment group, which received less food. The effect of the grading treatment on the initially-smaller families was not as apparent. The variance among family means is 39% lower in the graded cohort than in the control cohort. Letters identify families, and colored lines identify the same family across the two treatments.

The total variance in body size was 25% smaller in the graded group than in the control group, a significant difference based on the Fligner Killeen median test (P = 7.65e-05, chi-squared = 15.64; control var = 2.96, graded var = 2.23, n1 = 615, n2 = 556). The variance among the 15 family means in the graded group (Var = 0.39) was 39% smaller than in the control group (Var = 0.64) group, although this difference was not statistically significant via the Fligner Killeen test (P = 0.076, chi-squared = 40.51; n1 = 15, n2 = 15). This difference in statistical

significance is presumably owing to the small sample size of only 15 families in each group vs. n = 615 and 556 in the test using individual fish. Similarly, the percentage of the total variance that was distributed among families (ICC value) was smaller in the graded group (0.15) than in the control group (0.21), but with broadly overlapping 95% confidence values (Table 1, Fig 6).

Treatment	ICC	V_w	V_a	Lower CI	Upper CI
control	0.21	2.37	0.63	0.073	0.35
grading	0.15	1.90	0.34	0.040	0.27

Table 1. Intraclass correlation (ICC) values and variance components for control and grading treatments. V_w = component of variance within each family; V_a = component of variation among families; CI = 95% confidence interval.

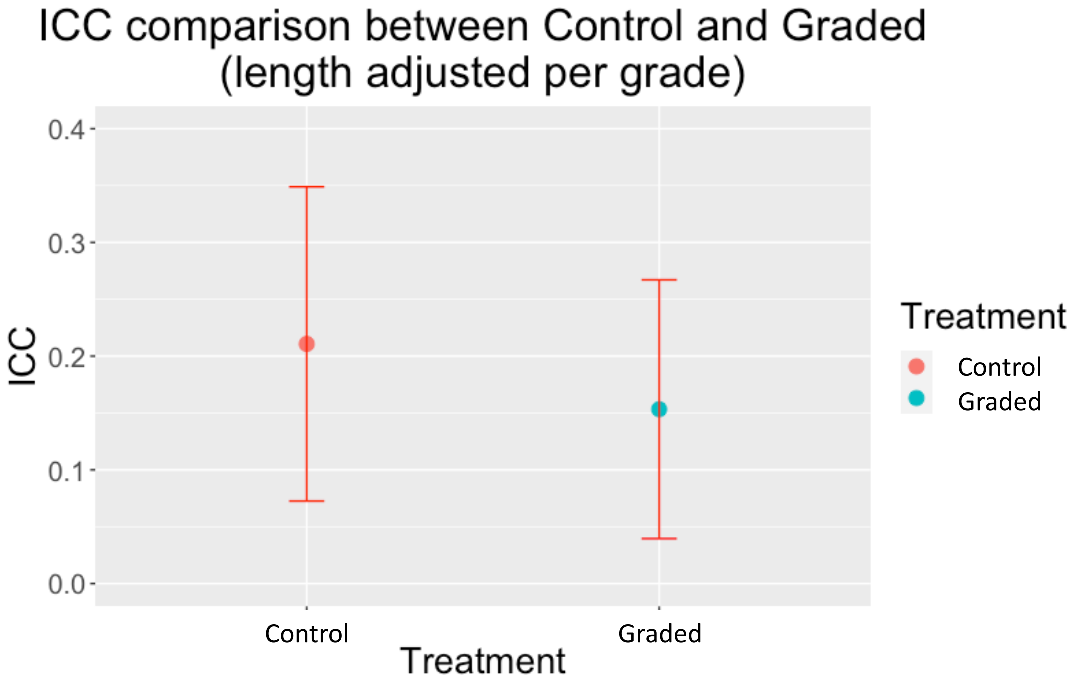

Fig 2. Intraclass correlation (ICC) values for control and grading treatments. The intraclass correlation is the fraction of total variance in size that is distributed among families. The broadly overlapping confidence intervals show the ICC was not significantly lower in the grading treatment.

Thus, the grading treatment did achieve a reduction in the nominal values of total variance in body size among all fish in the treatment, in the variance among family means (our

proxy for heritable variation in size), and in the among-family component of the total variance, although only the reduction in variance among individual fish was statistically significant.

Discussion

Grading fish into three groups and raising them with different growth targets produced a cohort that had a smaller total variance in size than the control cohort, which was reared under standard conditions. The variance among family means, and the percentage of total variance distributed among families, was also smaller in the graded cohort, although not significantly so. Nevertheless, a nominal 39% reduction in the variance among family mean size seems biologically interesting, as this variance should be a proxy for heritable variation in size that could be subject to selection.

The opportunity for selection can be interpreted as the extent of variation that could be subject to natural selection and is defined as the ratio of variation in fitness to the square of mean fitness ($I = (CV/100)^2$), and (Downhower, Blumer, and Brown 1987). As we consider size at release to be a component of fitness (predicts survival at sea), then in this case we estimate a 20.90% reduction in the opportunity for selection among individuals, and a 35.50% reduction in the opportunity for selection among families (CV among individuals = 7.98 and 8.97, and among families = 3.34 and 4.16 for control and graded, respectively). Thus, the grading treatment was successful in that it did seem to have the desired effect, although whether this would be enough to appreciably slow the rate of domestication selection is unknown at this point.

It is interesting that this reduction in variance was achieved almost entirely by holding back the growth of the initially-large grading group, and of the families that were overrepresented in that group. Relieving the initially-small families from competition with larger fish and raising them in low density tanks with high food availability did little to accelerate their

growth relative to the controls. Thus, we conclude that fish in the left-hand tail of the growth distribution are growing slowly due to inherent physiological or behavioral traits that limit their ability to take advantage of the hatchery setting, rather than to suppression by larger, more aggressive fish. Future research focused on what traits characterize those slow-growing fish might point to other environmental modifications one could make to accelerate their growth and further reduce the among-family variation. Until then, reduction in the opportunity for domestication selection that one can achieve by grading will probably be modest.

Grading and discarding small fish is commonly used in hatcheries to achieve more uniformly sized fish at the time of release (Meyer and Cassinelli 2020). Thus, implementing this variation of grading (separating initially-large and initially-small fish, and raising them separately) as a way to reduce variation in size at release might not be too difficult for hatcheries to implement. However, although grading and separate rearing was an effective way to slow the growth of the initially-large fish, there was little impact on the growth of the initially-smaller fish. While the grading treatment led to some reduction in the opportunity for selection, the average size of the grading group was also smaller than of the control group. As size is correlated to survival at sea (Clarke, Flesher, and Carmichael 2014; Osterback et al. 2014; Tipping 1986), this would predict fewer returns from the grading group, which would be undesirable in a production hatchery. However, grading might be appropriate in a conservation setting where creating the most wild-like fish possible is the goal, rather than producing the greatest number of returning fish. Grading could be most effective for conservation work if one can find a way to positively influence the growth of the initially-small fish along with reducing growth of the initially-large fish.

Acknowledgements

Thanks to the Oregon Department of Fisheries and Wildlife (ODFW), especially John Spangler and staff at the Siletz Falls trap facility, and to staff at the Oregon Hatchery Research Center and the Aquatic Animal Heath Lab for invaluable assistance in obtaining eggs and raising fish for these experiments.

Funding Statement

This work was funded by the ODFW through the Oregon Hatchery Research Center.

Data Availability Statement

All of the data generated and used in this study is available through Github, [https://github.com/obleym/Grading_Data_Obley]

References

1. Abbott, Jeremy C., and Lawrence M. Dill. 1989. “The Relative Growth of Dominant and Subordinate Juvenile Steelhead Trout (Salmo Gairdneri) Fed Equal Rations.” Behaviour 108 (1/2): 104–13.
2. Ahlbeck Bergendahl, I., S. Miller, C. Depasquale, L. Giralico, and V. A. Braithwaite. 2017. “Becoming a Better Swimmer: Structural Complexity Enhances Agility in a Captive-Reared Fish.” Journal of Fish Biology 90 (3): 1112–17. https://doi.org/10.1111/jfb.13232.
3. “ALSEA F H FALL CREEK, OREGON - Climate Summary.” n.d. Accessed March 20, 2024. https://wrcc.dri.edu/cgi-bin/cliMAIN.pl?or0145.
4. Biro, Peter A., Mark V. Abrahams, and John R. Post. 2007. “Direct Manipulation of Behaviour Reveals a Mechanism for Variation in Growth and Mortality among Prey Populations.” Animal Behaviour 73 (5): 891–96. https://doi.org/10.1016/j.anbehav.2006.10.019.
5. Biro, Peter A., Mark V. Abrahams, John R. Post, and Eric A. Parkinson. 2004. “Predators Select against High Growth Rates and Risk-Taking Behaviour in Domestic Trout Populations.” Proceedings. Biological Sciences 271 (1554): 2233–37. https://doi.org/10.1098/rspb.2004.2861.
6. Blouin, Michael S., Madeleine C. Wrey, Stephanie R. Bollmann, James C. Skaar, Ronald G. Twibell, and Claudio Fuentes. 2021. “Offspring of First-Generation Hatchery Steelhead Trout (Oncorhynchus Mykiss) Grow Faster in the Hatchery than Offspring of Wild Fish, but Survive Worse in the Wild: Possible Mechanisms for Inadvertent

Domestication and Fitness Loss in Hatchery Salmon." PloS One 16 (12): e0257407. https://doi.org/10.1371/journal.pone.0257407.

7. Branigan, Philip R., Kevin A. Meyer, and John D. Cassinelli. 2021. "Relative Cost and Post-Release Performance of Hatchery Catchable Rainbow Trout Grown to Two Target Sizes." Fisheries 46 (8): 357–71. https://doi.org/10.1002/fsh.10621.
8. Carlson, Stephanie M., and Todd R. Seamons. 2008. "A Review of Quantitative Genetic Components of Fitness in Salmonids: Implications for Adaptation to Future Change." Evolutionary Applications 1 (2): 222–38. https://doi.org/10.1111/j.1752-4571.2008.00025.x.
9. Christie, Mark R., Michael J. Ford, and Michael S. Blouin. 2014. "On the Reproductive Success of Early-Generation Hatchery Fish in the Wild." Evolutionary Applications 7 (8): 883–96. https://doi.org/10.1111/eva.12183.
10. Christie, Mark R., Melanie L. Marine, Rod A. French, and Michael S. Blouin. 2012. "Genetic Adaptation to Captivity Can Occur in a Single Generation." Proceedings of the National Academy of Sciences 109 (1): 238–42. https://doi.org/10.1073/pnas.1111073109.
11. Clarke, Lance R., Michael W. Flesher, and Richard W. Carmichael. 2014. "Hatchery Steelhead Smolt Release Size Effects on Adult Production and Straying." North American Journal of Aquaculture 76 (1): 39–44. https://doi.org/10.1080/15222055.2013.837127.
12. Downhower, Jerry F., Lawrence S. Blumer, and Luther Brown. 1987. "Opportunity for Selection: An Appropriate Measure for Evaluating Variation in the Potential for

Selection?" Evolution 41 (6): 1395–1400. https://doi.org/10.1111/j.1558-5646.1987.tb02476.x.

13. Edward D. Weber and Kurt D. Fausch. 2003. "Interactions between Hatchery and Wild Salmonids Instreams: Differences in Biology and Evidence for Competition." Canadian Journal of Fisheries and Aquatic Sciences 60:1018–36.
14. Feldhaus, Joseph W., Timothy L. Hoffnagle, and Richard W. Carmichael. 2016. "The Influence of Size at Release on Performance of Imnaha River Chinook Salmon Hatchery Smolts." North American Journal of Fisheries Management, March. https://www.tandfonline.com/doi/full/10.1080/02755947.2015.1128999.
15. Howe, Natasha S., Matthew C. Hale, Charles D. Waters, Sara M. Schaal, Kyle R. Shedd, and Wesley A. Larson. 2024. "Genomic Evidence for Domestication Selection in Three Hatchery Populations of Chinook Salmon, Oncorhynchus Tshawytscha." Evolutionary Applications 17 (2): e13656. https://doi.org/10.1111/eva.13656.
16. Huntingford, F. A. 2004. "Implications of Domestication and Rearing Conditions for the Behaviour of Cultivated Fishes." Journal of Fish Biology 65 (s1): 122–42. https://doi.org/10.1111/j.0022-1112.2004.00562.x.
17. Koch, Ilana J., and Shawn R. Narum. 2021. "An Evaluation of the Potential Factors Affecting Lifetime Reproductive Success in Salmonids." Evolutionary Applications 14 (8): 1929–57. https://doi.org/10.1111/eva.13263.
18. Lessells, C. M., and Peter T. Boag. 1987. "Unrepeatable Repeatabilities: A Common Mistake." The Auk 104 (1): 116–21. https://doi.org/10.2307/4087240.
19. Li, Hiram W., and Robert W. Brocksen. n.d. "Approaches to the Analysis of Energetic Costs of Intraspecific Competition for Space by Rainbow Trout (Salmo Gairdneri)."

Accessed July 31, 2024. https://onlinelibrary.wiley.com/doi/10.1111/j.1095-8649.1977.tb04126.x.

20. McMillan, John R., Brian Morrison, Nick Chambers, Greg Ruggerone, Louis Bernatchez, Jack Stanford, and Helen Neville. 2023. "A Global Synthesis of Peer-reviewed Research on the Effects of Hatchery Salmonids on Wild Salmonids." Fisheries Management and Ecology, July, fme.12643. https://doi.org/10.1111/fme.12643.
21. Meyer, Kevin A., and John D. Cassinelli. 2020. "The Effect of Raceway Grading on Return to Creel for Catchable-Sized Hatchery Rainbow Trout." North American Journal of Aquaculture 82 (2): 224–34. https://doi.org/10.1002/naaq.10149.
22. Nelson, R. J., T. D. Beacham, and M. P. Small. 1998. "Microsatellite Analysis of the Population Structure of a Vancouver Island Sockeye Salmon (Oncorhynchus Nerka) Stock Complex Using Nondenaturing Gel Electrophoresis." Molecular Marine Biology and Biotechnology 7 (4): 312–19.
23. Nicieza, A. G., and N. B. Metcalfe. 1999. "Costs of Rapid Growth: The Risk of Aggression Is Higher for Fast-Growing Salmon." Functional Ecology 13 (6): 793–800. https://doi.org/10.1046/j.1365-2435.1999.00371.x.
24. Obley, Miriam B., Ruth H. Milston-Clements, Jennifer A. Krajcik and Michael S. Blouin. " The effect of structural enrichment and increased water flow on the opportunity for domestication selection in hatchery-reared steelhead (Oncorhynchus mykiss)." Canadian Journal of Fisheries and Aquatic Sciences In Press
25. Osterback, Ann-Marie K., Danielle M. Frechette, Sean A. Hayes, Morgan H. Bond, Scott A. Shaffer, and Jonathan W. Moore. 2014. "Linking Individual Size and Wild and Hatchery Ancestry to Survival and Predation Risk of Threatened Steelhead

(Oncorhynchus Mykiss).” Canadian Journal of Fisheries and Aquatic Sciences 71 (12): 1877–87. https://doi.org/10.1139/cjfas-2014-0097.

26. Pasquet, Alain. 2018. “Effects of Domestication on Fish Behaviour.” In Animal Domestication. IntechOpen. https://doi.org/10.5772/intechopen.78752.

27. Petersson, Erik, and Torbjörn Järvi. 2000. “Both Contest and Scramble Competition Affect the Growth Performance of Brown Trout, Salmo Trutta, Parr of Wild and of Sea-Ranched Origins.” Environmental Biology of Fishes 59 (2): 211–18. https://doi.org/10.1023/A:1007645411586.

28. Roberts, L. J., J. Taylor, and C. Garcia de Leaniz. 2011. “Environmental Enrichment Reduces Maladaptive Risk-Taking Behavior in Salmon Reared for Conservation.” Biological Conservation 144 (7): 1972–79. https://doi.org/10.1016/j.biocon.2011.04.017.

29. Stephenson, Jeff J., Matt R. Campbell, Jon E. Hess, Chris Kozfkay, Andrew P. Matala, Megan V. McPhee, Paul Moran, et al. 2009. “A Centralized Model for Creating Shared, Standardized, Microsatellite Data That Simplifies Inter-Laboratory Collaboration.” Conservation Genetics 10 (4): 1145–49. https://doi.org/10.1007/s10592-008-9729-4.

30. Sundström, L. Fredrik, Erik Petersson, Johan Höjesjö, Jörgen I. Johnsson, and Torbjörn Järvi. 2004. “Hatchery Selection Promotes Boldness in Newly Hatched Brown Trout (Salmo Trutta): Implications for Dominance.” Behavioral Ecology 15 (2): 192–98. https://doi.org/10.1093/beheco/arg089.

31. Thodesen, Jørn, Barbara Grisdale-Helland, Ståle J. Helland, and Bjarne Gjerde. 1999. “Feed Intake, Growth and Feed Utilization of Offspring from Wild and Selected Atlantic Salmon (Salmo Salar).” Aquaculture 180 (January):237–46. https://doi.org/10.1016/S0044-8486(99)00204-5.

32. Thompson, Neil F., and Michael S. Blouin. 2015. “The Effects of High Rearing Density on the Potential for Domestication Selection in Hatchery Culture of Steelhead (Oncorhynchus Mykiss).” Canadian Journal of Fisheries and Aquatic Sciences 72 (12): 1829-.

33. Tipping, Jack. 1986. “Effect of Release Size on Return Rates of Hatchery Sea-Run Cutthroat Trout.” The Progressive Fish-Culturist 48 (3): 195–97. https://doi.org/10.1577/1548-8640(1986)48<195:EORSOR>2.0.CO;2.

34. Wallat, G. K., L. G. Tiu, H. P. Wang, D. Rapp, and C. Leighfield. 2005. “The Effects of Size Grading on Production Efficiency and Growth Performance of Yellow Perch in Earthen Ponds.” North American Journal of Aquaculture 67 (1): 34–41. https://doi.org/10.1577/FA04-003.1.

35. Waples, Robin S., Michael J. Ford, and Dietrich Schmitt. 2007. “Empirical Results of Salmon Supplementation in the Northeast Pacific: A Preliminary Assessment.” In Ecological and Genetic Implications of Aquaculture Activities, edited by Theresa M. Bert, 383–403. Dordrecht: Springer Netherlands. https://doi.org/10.1007/978-1-4020-6148-6_21.

36. Wright, Dominic, Reiichiro Nakamichi, Jens Krause, and Roger Butlin. 2006. “QTL Analysis of Behavioral and Morphological Differentiation Between Wild and Laboratory Zebrafish (Danio Rerio).” Behavior Genetics 36 (April):271–84. https://doi.org/10.1007/s10519-005-9029-4.

Click here to access/download
Supporting Information
Grading_Sup_Mat.xlsm
